# Preoperative Diagnosis of Gallbladder Agenesis: A Case Report

**DOI:** 10.7759/cureus.30753

**Published:** 2022-10-27

**Authors:** Alec Rivera, Dustin Williamson, German Almonte

**Affiliations:** 1 General Surgery, William Carey University College of Osteopathic Medicine, Hattiesburg, USA; 2 General/Trauma Surgery, Highland Community Hospital, Picayune, USA

**Keywords:** hida, endoscopic retrograde cholangiopancreatography (ercp), gallbladder ultrasound, non-visualized gallbladder, biliary dysfunction, biliary colic, sphincter of oddi dysfunction, agenesis of gallbladder

## Abstract

Gallbladder agenesis (GA) is a rare congenital malformation with less than 500 reported cases. Often an incidental finding intraoperatively or in postmortem autopsies, the first reported case dates back to 1701. We discuss the case of a 21-year-old female presenting with classic biliary symptoms who, on imaging, failed to have a visualizable gallbladder. Initial ultrasound (US) reported a non-visualized gallbladder with gallbladder contraction as a possible explanation. More advanced imaging allowed for the preoperative diagnosis, thus preventing an unpleasant intraoperative surprise. As imaging techniques continue to advance, unnecessary operations can hopefully be avoided, albeit a difficult diagnosis to make.

## Introduction

Gallbladder agenesis (GA) is a rare congenital malformation with less than 500 reported cases, which is often an incidental intraoperative or postmortem finding [1,2]. With an increased prevalence in females at 3:1, symptomatic cases generally present with classic biliary symptoms, as in our case [1-4]. The rarity and difficulty in diagnosing this disease radiologically often lead to unnecessary operative intervention. We present the case of a 21-year-old female who demonstrated typical symptoms of biliary dysfunction. Initial imaging reported a lack of visualization of the gallbladder; with the utilization of more advanced imaging techniques, we arrived at the diagnosis.

## Case presentation

A 21-year-old female with a medical history of premature birth and gastroesophageal reflux with no surgical history presented to her primary care physician (PCP) due to chronic worsening epigastric and right upper quadrant (RUQ) pain. The pain was achy, would worsen with every meal or drink, and was associated with lower abdominal cramping, nausea, and vomiting. Occasionally, it would radiate toward her back. Physical examination demonstrated diffuse abdominal pain, especially in the right upper quadrant (RUQ) and epigastric region; no signs of previous intra-abdominal surgical intervention were observed.

**Figure 1:**
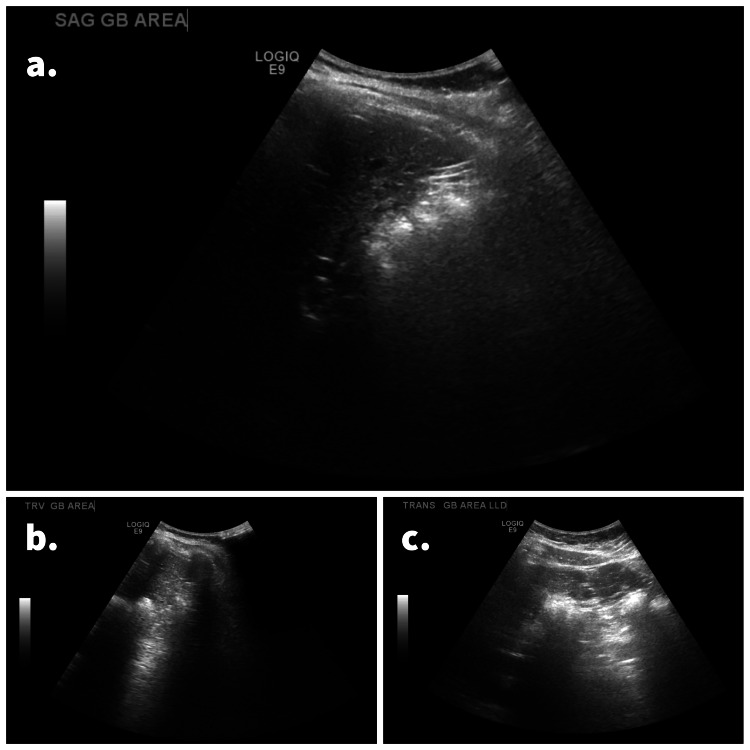
RUQ ultrasound. This was reported as a non-visualized gallbladder possibly due to a contracted gallbladder or stones filling the gallbladder leading to significant shadowing. (a) A sagittal view of the expected gallbladder location. (b and c) Alternative views of the anticipated gallbladder location. RUQ: right upper quadrant.

**Figure 2:**
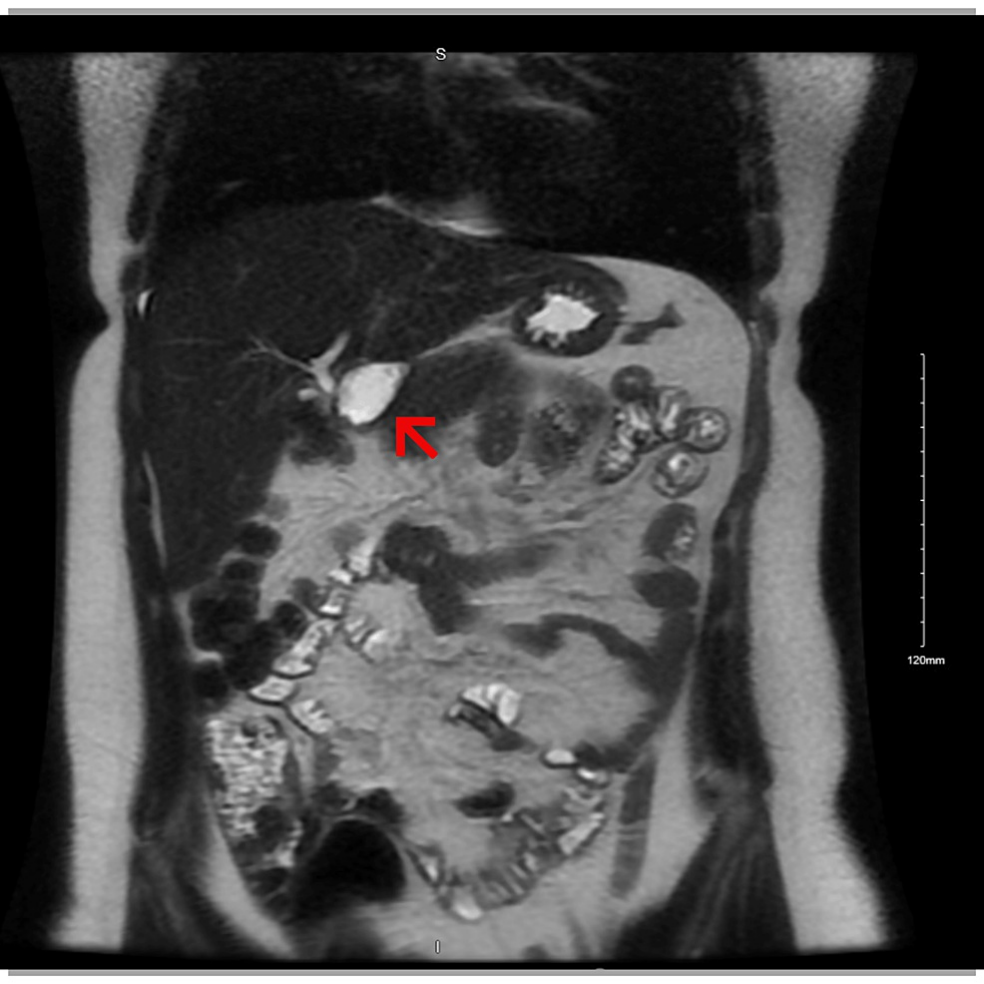
Coronal view of abdominal MRI with contrast. The structure is considered as a possible left-sided gallbladder (arrow), but as seen in Figure 5, this is more likely consistent with the post-pyloric duodenum.

**Figure 3:**
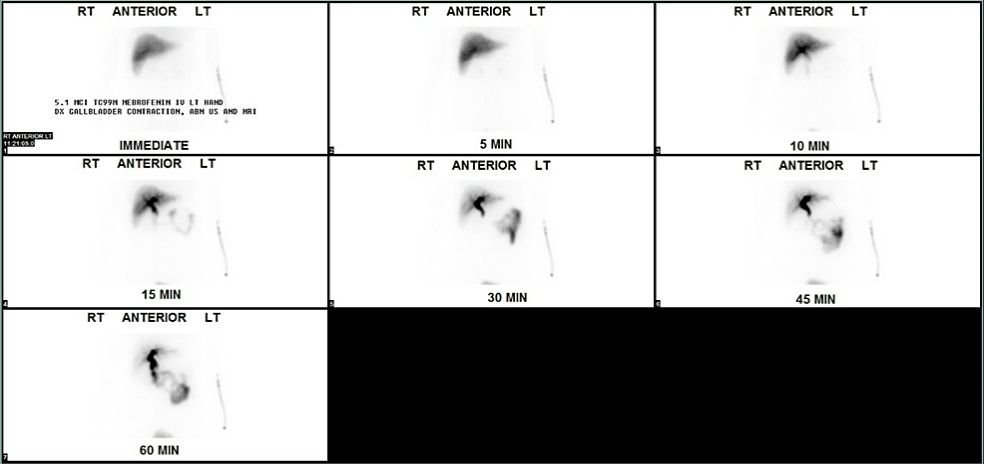
HIDA scan demonstrating no visualization of the gallbladder. HIDA: hepatobiliary iminodiacetic acid.

**Figure 4:**
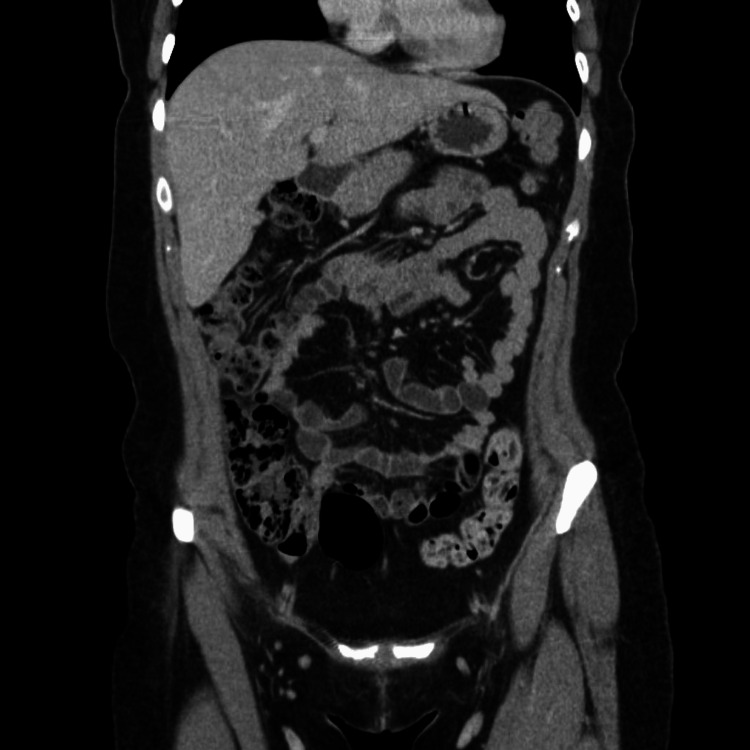
Coronal view of abdominal and pelvic CT with contrast (reported as a non-visualized gallbladder). There appears to be no sign of previous surgical intervention such as surgical clips. Initial review led to a left-sided gallbladder as a differential, but when correlated with a cross-sectional view (Figure 5), this appears to be the first part of the duodenum. CT: computed tomography.

**Figure 5:**
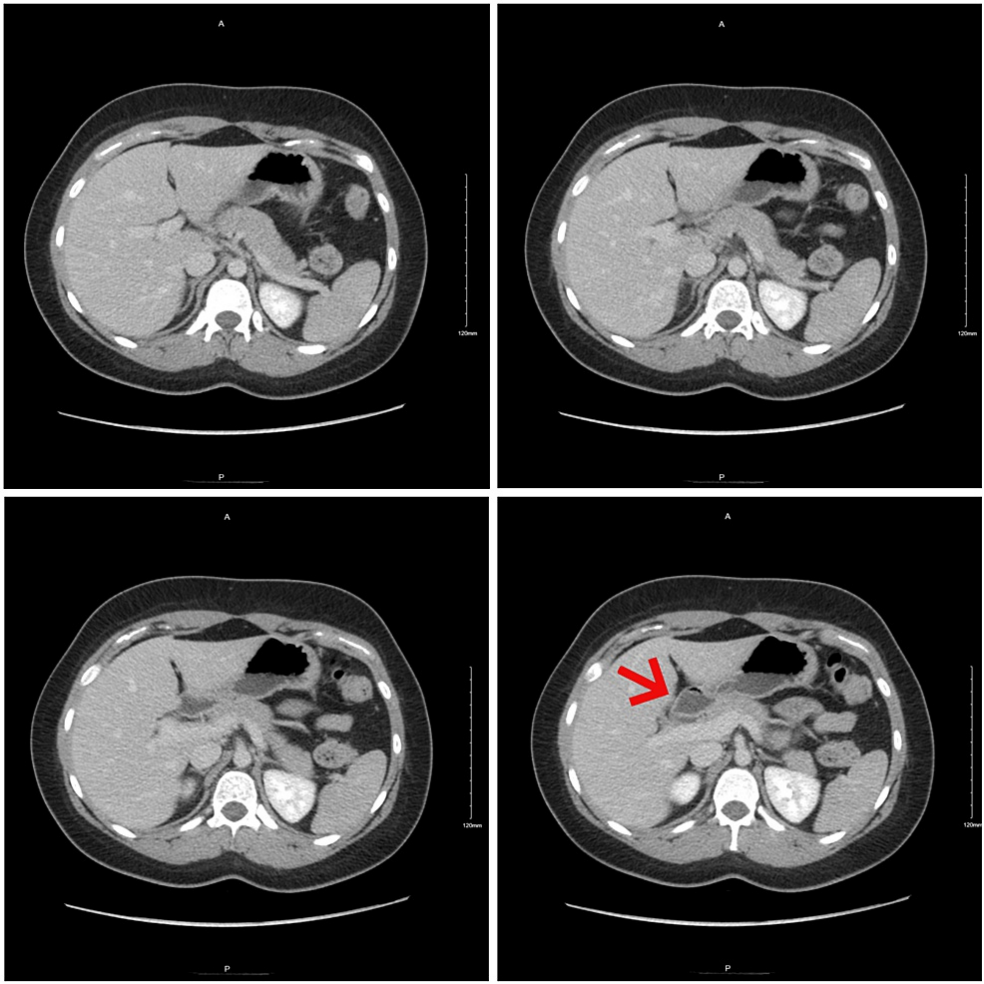
Abdominal and pelvic CT with contrast. We can see the structure thought possibly representing a left-sided gallbladder, which is more consistent with the post-pyloric duodenum (arrow). CT: computed tomography.

**Figure 6:**
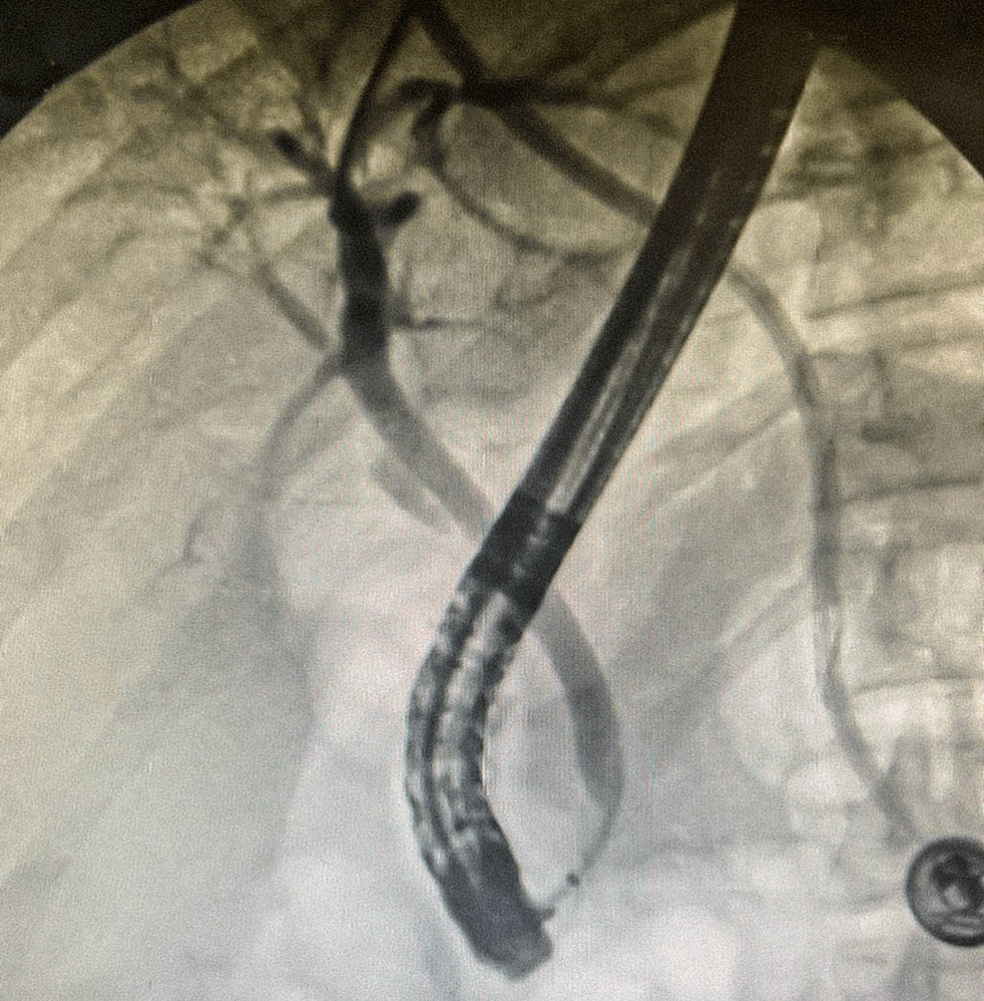
Intraoperative ERCP fluoroscopic images demonstrating no cystic duct or gallbladder. ERCP: endoscopic retrograde cholangiopancreatography.

RUQ ultrasound (US) ordered by the patient’s primary care physician demonstrated “no visualization of the gallbladder, which could be related to either a contracted gallbladder or possibly stones filling the gallbladder causing shadowing” (Figure 1a-1b). A subsequent MRI with and without contrast (Figure 2) and hepatobiliary iminodiacetic acid (HIDA) scan (Figure 3) were also unable to identify the gallbladder. A left-sided gallbladder was added as a differential diagnosis following the abdominal MRI (Figure 2), although, as seen in subsequent imaging, this was more consistent with the first part of the duodenum (Figure 5). Prior to further imaging, the patient’s mother was contacted to clarify her neonatal course. Per the patient’s mother, the patient was born premature and spent several weeks in the neonatal intensive care unit (NICU). Her clinical course was complicated by hyperbilirubinemia and required ultraviolet (UV) light therapy treatment but did not necessitate surgical intervention, nor did the patient ever receive any surgical operation. An abdominal computed tomography (CT) with IV contrast again was unable to confidently visualize the gallbladder (Figures 4-5). Following the inconclusive results of the CT and US and extensive deliberation with radiology, endoscopic retrograde cholangiopancreatography (ERCP) was decided on as the next step (Figure 6). The subsequent ERCP demonstrated a moderate amount of biliary sludge, for which a sphincterotomy was performed. This was likely resultant from a sphincter of Oddi dysfunction, thus explaining the patient’s symptoms. At one-month follow-up of ERCP with sphincterotomy, there was a complete resolution of the patient’s symptoms.

## Discussion

Gallbladder development starts in the four-week-old embryo, forming from a hepatic diverticulum, the bud of the liver. This bud is the primordium of the liver, ventral pancreas, and biliary system. The bud separates into rostral and caudal segments, with the caudal eventually forming the gallbladder. The inappropriate migration of the caudal segment leads to an ectopic gallbladder, but failure in development leads to the agenesis of the gallbladder [5,6].

Initially reported by Bergman in 1701, GA is a very rare congenital malformation [7]. When diagnosed during surgery, it is predominantly seen with a female-to-male ratio of 3:1. GA was previously categorized into three different groups: 1) GA with multiple fetal anomalies, 2) asymptomatic GA, and 3) symptomatic GA [2,3,8,9]. In 2015, a new classification system was proposed: type I (symptomatic) and type II (asymptomatic). Type I is further subdivided into Ia (with fetal malformations) and Ib (without fetal malformations). Type Ib is the most prevalent [5,6]. Symptoms classically present as usual biliary symptoms (right upper quadrant pain, dyspepsia, and fatty food intolerance), as in our case and others reported* *[1-4,10]. Due to an extremely low incidence of 0.01%-0.075%, GA is often overlooked or misdiagnosed [3,8,11].

The initial US can often be misleading, and a contracted gallbladder is often reported as the reason for no visualization [1,8,9,12-14]. This often results in surgical intervention, and the inability to expose the critical view of the triangle of Calot. The continued manipulation during the search for these structures can lead to an increased risk of iatrogenic injury. Although there is a paucity in the literature for a standardized diagnostic approach in patients with suspected GA or a non-visualized gallbladder on US, recommendations are similar. In the event of an inconclusive US, further imaging such as CT, ERCP, and magnetic resonance cholangiopancreatography (MRCP) before operative management is recommended [2,6,13]. This will not eliminate all operations, as GA can be difficult to diagnose even with advanced imaging studies, but it will likely decrease the incidence of premature operative intervention.

As with the diagnostic approach, there is limited information on treatment options for patients with symptomatic GA. It has been noted, though, that patients who have been found to have GA intraoperatively have symptom resolution following the operation. The mechanism for this resolution is not fully understood but has been theorized to be a result of periportal and right hypochondrial adhesion lysis [2-4,9]. There has also been some noted success with smooth muscle relaxants and analgesics if the pain continued throughout the postoperative period [2,15]. This therapy or a sphincterotomy could be beneficial if the symptoms presented due to a sphincter of Oddi dysfunction, as in our patient. More studies will be needed for the best treatment options to be recommended.

It is also of importance to note the probable genetic component of gallbladder agenesis. While this is still somewhat controversial, a case report involving monozygotic twins is most supportive of a genetic component. The report presented the case as a symptomatic patient, whose diagnosis of gallbladder agenesis prompted screening of her asymptomatic twin also demonstrating gallbladder agenesis [16]. Screening may not be of significant benefit in asymptomatic relatives, but should they become symptomatic, a family history of gallbladder agenesis should trigger a more meticulous investigation with a lower threshold for pursuing advanced imaging.

## Conclusions

Gallbladder agenesis continues to be a challenging diagnosis to arrive at prior to surgical intervention. Although there is no dedicated diagnostic approach, further imaging should be pursued when initial US is unrevealing, especially in instances when a family history of gallbladder agenesis is present. We believe that with continued advances and improved availability of preoperative imaging, unnecessary and potentially harmful operations can be avoided so long as we keep this rare condition in our differentials.
